# Chronic over-nutrition and dysregulation of GSK3 in diseases

**DOI:** 10.1186/s12986-016-0108-8

**Published:** 2016-08-04

**Authors:** Xunxian Liu, Zemin Yao

**Affiliations:** Department of Biochemistry, Microbiology and Immunology, Ottawa Institute of Systems Biology, University of Ottawa, 451 Smyth Road, Ottawa, ON K1H 8M5 Canada

## Abstract

Loss of cellular response to hormonal regulation in maintaining metabolic homeostasis is common in the process of aging. Chronic over-nutrition may render cells insensitive to such a hormonal regulation owing to overstimulation of certain signaling pathways, thus accelerating aging and causing diseases. The glycogen synthase kinase 3 (GSK3) plays a pivotal role in relaying various extracellular and intracellular regulatory signals critical to cell growth, survival, regeneration, or death. The main signaling pathway regulating GSK3 activity through serine-phosphorylation is the phosphoinositide 3-kinase (PI3K)/phosphoinositide-dependent kinase-1 (PDK1)/Akt relay that catalyzes serine-phosphorylation and thus inactivation of GSK3. In addition, perilipin 2 (PLIN2) has recently been shown to regulate GSK3 activation through direct association with GSK3. This review summarizes current understanding on environmental and nutritional factors contributing to GSK3 regulation (or dysregulation) through the PI3K/PDK1/Akt/GSK3 axis, and highlights the newly discovered role that PLIN2 plays in regulating GSK3 activity and GSK3 downstream pathways.

## Background

Glycogen synthase kinase 3 (GSK3) is a serine/threonine protein kinase [1] and catalyzes phosphorylation of perhaps more than 100 substrates [2]. A unique feature associated with GSK3 regulation is that the enzyme is “constitutively” activated (i.e. always in the “on” stage”) in cells [3], probably due to a recently identified phosphorylation of GSK3 catalyzed by protein kinase (PK) Cζ [4]. Physiological inhibitors of GSK3 include phosphoinositide 3-kinase (PI3K)/phosphoinositide-dependent kinase-1 (PDK1)/Akt relay [5], as well as the newly identified lipid-binding protein perilipin 2 (PLIN2), also known as adipose differentiation-related protein (ADRP) [6]. In the PI3K/PDK1/Akt relay, Akt is activated by PDK1 [7], and PDK1 in turn is activated by PI3K-generated 3-phosphorylated phosphoinositides [8]. In this review, the PI3K/PDK1/Akt axis involved in GSK3 inhibition is abbreviated as PI3K/Akt pathway. Because PI3K activities are modulated by a variety of factors, including hormones, lipid, drugs, food components and food metabolic products through their respective receptors [9], the PI3K/Akt/GSK3 axis represents a major regulatory pathway that relays extracellular and intracellular signals critical to cell growth, survival, regeneration, or death.

It is thus generally believed that negative regulation of GSK3, through PI3K/Akt-mediated phosphorylation, keeps GSK3 activity at “off” or “low” stages [2]. Under disease conditions, either genetic abnormalities or unhealthy environmental factors (such as life style, habits, psychology, and medication) can break cellular homeostasis and lead to increased GSK3 activity and/or unbridled GSK3 activation. Overly activated GSK3 in disease stages, interestingly, can occur when PI3K/Akt is either repressed (resulting from exogenous or endogenous inhibitors or lack of stimuli) or over-stimulated (resulting from high levels of stimuli). High GSK3 activity has been implicated in Bipolar Disorder [9], Parkinson’s disease [1], Alzheimer’s disease, and Type 2 diabetes (T2D) [10]. Locally low GSK3 activity yet systemically high GSK3 activity may associate with cancer [5, 6, 10]. Moreover, GSK3 can promote inflammation [11] and has been suggested to play a role in aging and age-related macular degeneration [5]. Many of these chronic abnormalities are considered as degeneration diseases. High GSK3 activity perhaps is also linked with several other chronic diseases, such as acquired immune deficiency syndrome (AIDS), cardiovascular diseases (CVD), liver diseases, lung diseases, and renal failure, which are associated with age, inflammation, and/or T2D [5, 6].

In addition to the PI3K/Akt/GSK3 axis, the GSK3 activity can also be attenuated by PLIN2 via its direct binding to GSK3 [6]. Serine phosphorylation of GSK3 (pSGSK3), catalyzed by Akt, renders GSK3 inactivation [5], whereas tyrosine phosphorylation of GSK3 (pYGSK3) results in an active form of the enzyme. GSK3 can be serine-phosphorylated by a number of kinases, including PKA [12], PKB (Akt) [5], and PKCα [13], even though in most studies only the Akt activity is measured. The increase in GSK3 activity can be achieved through at least three mechanisms, (i) tyrosine-phosphorylation (pYGSK3) [6], (ii) dissociation from PLIN2 [6], and (iii) lowered serine-phosphorylation (pSGSK3) [5]; all of which exert similar impacts on cell growth/survival [5, 6]. High GSK3 activity is invariably associated with increased cell apoptosis [14–16], causing abnormal cell death as well as cell regeneration [5].

High intracellular lipid levels for different time periods appear to modulate PLIN2 binding to GSK3 and to GSK3 substrates, thus impacting the GSK3 activity and GSK3 downstream pathways, in turn, cell survival/growth [6]. High and low GSK3 activities in the body may underlie the biological mechanisms due to hyperlipidemia and obesity [6]. Elevated intracellular lipid concentrations mimic the body cells under hyperlipidemia and obesity circumstances [6], and obesity is closely associated with increased risks of chronic diseases, including Alzheimer’s disease [17], cancer, CVD, T2D [18], and hyperlipidemia [17].

Loss of cellular response to hormonal regulation in maintaining metabolic homeostasis is common in the process of aging [5]. Chronic over-nutrition creates an environment that overly stimulates certain signaling pathways (e.g. PI3K/Akt), which can render cells insensitive to hormonal stimulation. This “overstimulation-induced insensitivity” phenomenon is commonly present in almost all of the metabolic disorders. This review focuses on overstimulation-induced insensitivity of GSK3, particularly on the signaling pathways that negatively modulate GSK3 activity and on the environmental and nutritional factors contributing to GSK3 regulation through the PI3K/PDK1/Akt/GSK3 axis. Current understandings on the newly discovered role that PLIN2 plays in regulating GSK3 activity and GSK3 downstream pathways are also highlighted. Although changes in the PI3K/Akt activities may also crosstalk to PLIN2-mediated GSK3 regulation, there is no such information reported in the literature. Therefore, the two aspects of GSK3 regulation are presented separately in this review. The well-described negative regulation of GSK3 activity by the Wnt signaling pathway is not discussed in this review.

## Regulation of GSK3 through phosphorylation/de-phosphorylation

Because GSK3 is constitutively activated [3], regulation of GSK3 is achieved mainly through the inhibitory serine-phosphorylation; namely, phosphorylation of serine-21 in GSK3α and phosphorylation of serine-9 in GSK3β in mammals. GSK3 is connected to extracellular environment primarily via PI3K/Akt, and thus maintenance of those kinase activities is essential for GSK3 inhibition. Activation of PI3K is achieved by various types of cell receptor signaling that in succession is modulated by alteration of environmental and/or genetic factors.

Although normal repression of GSK3 activity is controlled by consecutive PI3K/PDK1/Akt activations, overstimulation of receptors, the upstream of PI3K under many disease conditions can cause insensitivity of the PI3K/Akt/GSK3 pathway and result in uninhibited GSK3 activity [5]. For instance, long-term treatment with hormones that are members of the growth hormone family, such as vascular endothelial growth factor (VEGF) can cause GSK3/Akt/PI3K insensitivity and activate GSK3 in cultured cells [5], whereas repeatedly administrating growth hormone, approximately equating to long-term hormone treatment is often associated with adverse effects such as diabetes and glucose intolerance in humans [19], which is attributable to possible high GSK3 activity. Likewise, persistent existence of a stimulus, such as high expression of interleukin 17 receptor C (IL17RC) in eyes and peripheral blood cells in age-related macular degeneration, is also associated with increased GSK3 activity [5, 20]. Endogenous ligands such as thyroxine [21] and growth hormone can also activate Akt and PI3K/Akt pathway [22, 23]. Decrease in sensitivity towards such ligands in aged cells [19, 21] is probably associated with diminished PI3K/Akt activation, which in turn contributes to uncontrolled GSK3 activity [5].

Therefore, release of GSK3 activity, a hallmark of PI3K/Akt/GSK3 pathway insensitivity can happen under disease conditions as a consequence of any of the following events: (i) overstimulation of PI3K/Akt, (ii) reduction of using native PI3K/Akt stimuli, and (iii) inhibition of PI3K/Akt.

Animal and cell culture studies combined have suggested that release of GSK3 activity can occur under different stages of PI3K/Akt/GSK3 pathway insensitivity. Based on immunoblot analysis of the phosphorylation status of the kinases, we have postulated that there are four stages (stage 0 through stage 3) of PI3K/Akt/GSK3 insensitivity [5]. Of those, the overstimulation-induced GSK3 insensitivity and activity are developed stage-wise, i.e. GSK3 insensitivity occurs first, which precedes Akt insensitivity and in turn PI3K insensitivity [5]. In stage 1, high GSK3 activity coincides with high PI3K and Akt activities; in stage 2: high GSK3 activity occurs where PI3K activity is high yet Akt activity is unchanged or low; in stage 3, high GSK3 activity coexists with low PI3K and Akt activities (Table 1). Thus, the higher the stage is, the more damages of the kinases sensitivity; moreover, the longer stimulus of the same system, the higher stage of the kinases insensitivity [5], which suggests that the highest stage can be reached in any system as long as a stimulation lasts sufficiently long. The overstimulation-induced insensitivity of PI3K/Akt/GSK3 can be pathway specific, as for example, IL17RC overexpression has little impact on the sensitivity of extracellular signal-regulated kinases (ERK) or Wnt signaling [5]. At stage 0, the kinases do not lose their sensitivity for normal regulation of phosphorylation, despite manifestation of diseases such as insulin resistance [24].

**Table 1:** Stages of the kinases insensitivity under disease conditions

Stage	Model system	PI3K activity	Akt activity	GSK3 activity	Insensitive kinase	Phenotype
0	Male Sprague Dawley (SD) Rats	High	High	Low	None	Early insulin resistance
1	Human retinal pigment epithelial cells (HRPE) treated with VEGF	High	High	High	GSK3	Low growth
2	Human monocytes overexpressing IL17RC or treated with VEGF	High	Unchanged or low	High	GSK3, Akt	Low growth
3	HRPE overexpressing IL17RC	Low	Low	High	GSK3, Akt, PI3K	Low growth

## Regulation of GSK3 through PLIN2

The perilipin (PLIN) family consists of a group of cytoplasmic proteins with sequence homology and characteristic binding to cytosolic lipid droplets [25]. A typical PLIN protein consists of a two-domain structure; the N-terminal lipid-binding domain and the C-terminal α-helix bundle, resembling some of the exchangeable apolipoproteins [26, 27]. While some PLIN proteins (e.g. PLIN1 and PLIN2) appear exclusively in association with lipid droplets, other PLIN proteins (e.g. PLIN3 or Tip47) can bind to subcellular organelles in addition to lipid droplets [28]. Mechanisms that regulate the PLIN protein partitioning between lipid droplets and organellar membranes are not defined.

Although it is generally believed that PLIN proteins are important for the metabolism (especially catabolism) of intracellular lipids [29], the exact role that PLIN plays in cellular lipid homeostasis remains largely unclear. Attempts were made to determine PLIN2 function using genetic manipulation approaches, such as antisense oligo [30], gene-knockout [31, 32], or siRNA in mice [33] or cultured cell lines [6]. Although data obtained from gene-knockout studies were confounded by the presence of a truncated PLIN2 segment in the mouse model [34], it is apparent that PLIN2 depletion in mice, even though the ablation may not be complete, is associated with amelioration of diet-induced hepatosteatosis, obesity, and adipocyte inflammation [32]. On the other hand, forced overexpression of PLIN2 in macrophages [35], hepatic stellate cells [36], HEK293 cells [37], or skeletal muscle C2C12 cells [33] could result in increased cytosolic lipid droplet content. The increase in cellular lipids, upon PLIN2 expression, cannot be predominantly attributed to increased lipid synthesis; rather, it is likely due to decrease in lipid turnover.

A potential role of PLIN2 overexpression in glucose uptake has been recently demonstrated using transfected mouse fibroblast L cells and differentiated 3T3-L1 adipocytes [38]. In these cells, a negative correlation between PLIN2 expression and glucose uptake was observed; thus, overexpression of PLIN2 in these cells results in markedly decreased glucose uptake, whereas PLIN2 knock-down is associated with manifold increase in glucose uptake [38]. The exact mechanism whereby PLIN2 expression could attenuate cellular glucose uptake is uncertain. It was assumed that PLIN2 might sequester SNAP27, a protein component of the SNARE complex that is required for the glucose transporter 1 trafficking to and from the plasma membranes [38]. Overexpression of PLIN2 alleviates insulin resistance in skeletal muscle cells [33].

We have recently obtained experimental evidence that PLIN2 is a GSK3-associated protein playing an obligatory role in the Wnt/Frizzled pathway, probably through acting as an intermediate between Dishevelled 2 (Dvl2) and the axin/GSK3β/β-catenin complex (AGβC) [6]. In 3T3-L1 and HEK293 cells, PLIN2 is required for Wnt-regulated disruption of axin/GSK3 complexes; upon Wnt-3α stimulation, the association of Dvl2/PLIN2 is decreased and concomitantly the association between AGβC and PLIN2 is increased within 15–30 min [6]. Importantly, this PLIN2-dependent AGβC disassembly appears to be independent of pSGSK3 levels, because its levels are unchanged upon Wnt stimulation [6]. The effect also appears to be specific to PLIN2, because silencing PLIN3 has no effect on Wnt-induced β-catenin stability [6]. The role played by PLIN2 as a relay between Dvl2 and AGβC in the canonical Wnt signaling was further authenticated by experiments in which Gα_o/q_ were silenced; silencing Gα_o_ and Gα_q_ abolished the Wnt-decreased Dvl2/PLIN2 association and Wnt-increased GSK3β/PLIN2 association [6].

It is known that Wnt stimulation can inhibit GSK3-mediated β-catenin phosphorylation through either disruption or else alteration of the AGβCs [39]. However, disassembly of the AGβCs is not associated with changes in GSK3 serine-phosphorylation. For instance, Wnt-induced, PLIN2-dependent AGβC disassembly in 3T3-L1 and HEK293 cells, as mentioned above, is unrelated to changes in pSGSK3 levels [5]. It is also known that β-catenin phosphorylation by the activity of GSK3 coupled to the AGβC is not controlled by the GSK3 serine-phosphorylation mechanism [2].

PLIN2 expression levels also exert an impact on cell growth; forced expression of PLIN2 in 3T3-L1 cells results in accelerated cell growth, which is associated with increased expression of GSK3 substrates including β-catenin, CCAAT enhancing binding protein α (c/EBPα), c-Myc, and cyclin D1 [6]. Conversely, silencing PLIN2 in the cells leads to a reduced expression of these GSK3 substrates. PLIN2 depletion also results in dropped pSGSK3 levels, indicative of attenuated GSK3 inhibition [6].

Showing that temporary PLIN2 depletion can decrease Wnt signaling including stabilization of β-catenin, a co-transcription factor, and expression of transcription activators (c/EBPα and c-Myc) and a cell-survival factor (cyclin D1), the above cell culture studies [6] suggest a developmental role of PLIN2, since regulated Wnt-signaling is essential for development [39]. The decreased cell growth/survival upon PLIN2 depletion is linked with decreased expression of the aforementioned substrates and increased GSK3 activity [6], which are known to induce cell apoptosis [14–16]. These cell culture PLIN2 silencing data apparently are in discord with the PLIN2 knockout mouse data, because homozygotes for a targeted PLIN2 mutation did not display discernible growth retardation [32]. It was found that a truncated PLIN2 protein, representing the C-terminal 80 % of the full-length PLIN2, was expressed in some tissues of the PLIN2 knockout mice [34]. The residual expression of this truncated PLIN2 protein (missing a region encoded by exons 2 and 3 of murine PLIN2) may still support normal mouse development, whereas animal models entirely devoid of PLIN2 may not survive in vertebrates [6, 31].

It has been well documented that cellular PLIN2 concentration is positively correlated with intracellular lipid contents [40]. Thus, treatment of cells with fatty acids, such as oleic acid, to stimulate cytosolic lipid droplet formation invariably generates increased cellular PLIN2 concentrations [40]. Remarkably, treatment of cells with oleic acid not only attenuated the Wnt-3α-induced associations between PLIN2 and AGβC components, in the face of elevated PLIN2 concentrations, but also inhibited β-catenin/T-Cell Factor (TCF) signaling [6]. Presumably, PLIN2 in oleic acid-treated cells is sequestered by cytosolic lipid droplets via PLIN2 strong lipid-binding affinity and therefore is unable to participate in mediating Wnt signaling [6]. The mechanism of oleic acid treatment suppressing Wnt-signaling is further confirmed by the treatment causing reduction of Wnt-induced AGβC/PLIN2 associations, whereas the treatment generates low levels of Dvl2/PLIN2 association similar to that under mere Wnt treatment [6].

Obesity increases the risks of many chronic diseases, including T2D, Alzheimer’s disease, cancer, CVD and hyperlipidemia [17, 18]. Diet-induced obesity and hyperlipidemia are often revealed by increases of both extracellular and intracellular lipid contents in human body [33, 41]. Data obtained from cell culture studies [6] suggest that the effects of intracellular lipid on GSK3 activity are time-dependent. Thus, the short-term oleic acid treatment actually significantly stimulates GSK3/PLIN2 association, suggesting that lower GSK3 activity at this stage raises the expression of c-Myc and cyclin D1 as well as cell growth/survival [6]. These data are consistent with studies showing that the cytosolic lipid droplets enhance TCF activity and carcinogenesis in colorectal cancer stem cells [42]. The short-term oleic acid treatment engenders intensified associations of GSK3/PLIN2 and PLIN2/GSK3 substrates at least in one cell line studied [6]. Thus, the increase in cell growth, upon short-term oleic acid treatment, is likely due to low GSK3 activity and high expression of c-Myc and cyclin D1, a Wnt-like effect mediated by PLIN2 [6]. These studies support the possibility that low GSK3 activity increases the risks of cancer [10].

On the other hand, in long-term oleic acid treatment, GSK3/PLIN2 association is lowered but GSK3 activity is increased. Thus, under this condition, association of GSK3/PLIN2 is inversely related to GSK3 activity that inhibits the expression of GSK3 substrates (e.g. c-Myc, cyclin D1, and insulin receptor substrate 1 (IRS1)) and cell growth/survival [6]. Presumably, dissociation of GSK3/PLIN2 may allow GSK3 to contact its substrates and synergistically to augment GSK3 activity in addition to lipid-induced pYGSK3 activity [6]. In this case, increase in intracellular lipids is functionally equivalent to PLIN2 depletion, bringing in increased degradation and/or phosphorylation of GSK3 substrates.

High levels of GSK3 activity under long-term oleic acid treatment conditions are associated with decreased c-Myc and cyclin D1 expression. Consequently, the cell growth/survival is reduced [6]. The influence of oleic acid treatment on GSK3 activity suggests a potential regulatory role of PLIN2 during the interplay of intracellular lipid metabolism and cell functionality, which may support a biological mechanism of diseases risks associated with increased hyperlipidemia and obesity. Indeed, high levels of GSK3 activity are detected in Alzheimer’s disease and T2D [10], and increased apoptosis has been observed [14–16]. An unrestricted high-fat diet generates increased GSK3 activity in the brain of a mouse model for Alzheimer’s disease study [43]. Detection of high levels of GSK3 activity in T2D [10] is probably linked with GSK3-mediated IRS1 phosphorylation [44] and degradation [45]. In this regard, the discovery that PLIN2 can interact with IRS1 and that PLIN2 expression exerts a major impact on IRS1 expression [6] is of particularly important significance because IRS1 is also a GSK3 substrate. Therefore, under long-term oleic acid treatment conditions, both high GSK3 activity and dissociation of PLIN2/IRS1 are suggested to promote IRS1 degradation [6], which is a contributing factor to T2D.

To sum up, PLIN2 mediates GSK3 activity in short-term and long-term oleic acid treatment conditions. If the in vitro cell culture data can be extrapolated to in vivo situation such as hyperlipidemia/obesity (since GSK3 and PLIN2 are ubiquitously expressed), both acute and chronic lipid effects may generate over-suppressed and uncontrolled GSK3 activity, potentially causing divergent damages [5, 6, 10]. Although the mode of GSK3 regulation differs between the PI3K/Akt-mediated mechanism (i.e. serine-phosphorylation) and lipid/PLIN2-mediated mechanism (i.e. tyrosine-phosphorylation and PLIN2/GSK3 interaction) [5, 6], the released GSK3 activity exerts similar impacts on cell growth/survival, which is suggested to have important effects on the initiation and progression of chronic diseases [5].

## Dietary and pharmacological considerations that influence GSK3 regulation

The PI3K/Akt pathway relays a vast number of extracellular signals, through their interaction with cell surface receptors (e.g. cytokine receptors, integrin, receptors of tyrosine kinase (RTK), and G-protein coupled receptors (GPCR)), to GSK3. Nutrients and drugs that either activate or antagonize the PI3K/Akt pathway will inevitably exert an impact on GSK3, and as discussed above, often lead to unchecked GSK3 activation, either through PI3K/Akt or else PLIN2.

Nutrients that affect PI3K/Akt pathway include water, proteins, carbohydrates, and fats (Table 2). Hypo-osmotic [46–48] or hyper-osmotic stresses [49–52] associated with disorders in water homeostasis are known to involve the PI3K/Akt signaling. Non-denatured and denatured proteins can activate PI3K/Akt. Branched-chain amino acids presented in cow milk are highly insulinotropic and a potent activator of PI3K/Akt [53–55]. Increased protein intake causes negative calcium balance in the body [56]. Excess dietary proteins produce a great amount of acids, mostly in the forms of sulfates and phosphates [57]. Magnesium sulfate, fucosylated chondroitin sulfate [58, 59], and heparan sulfates [60] all play a role in PI3K/Akt pathway activities.

**Table 2:** Water, proteins or fats affect PI3K/Akt and/or GSK3 activities

Nutrient	Model system	Observed effects	Ref. (model)
Water			
Hypo-osmotic stress	Human embryonic kidney cells, mouse osteoblast, human thyroid cancer cells, HRPE, human monocytes and human neuroblastoma.	Hypo-osmotic pressure induces calcium influx that mediates PI3K and p53 activation, resulting in cell apoptosis, which involves high GSK3 activity due to overstimulation.	[5, 15, 46–48]
Hyper-osmotic stress	Monkey kidney cells, HeLa cells, human or mouse melanoma cells, HRPE, human monocytes.	Despite inducing the p21-activated serine-threonine kinase, requiring PI3K activation, within 30-min, hyper-osmotic stress suppresses melanin production that also requires PI3K activation, for days of the treatment, suggesting overstimulation of the PI3K/Akt pathway.	[5, 49–52]
Proteins			
Non-denatured protein	Male SD rats, human embryonic kidney cells.	Branched-chain amino acids in cow milk are highly insulinotropic and a potent activator of PI3K/Akt.	[53, 54]
Denatured proteins	Rat muscle cells.	Increase PI3K.	[55]
Excess protein	Adult women, rats with intestinal ischemia-reperfusion injury, T2D mice.	Raise calcium excretion; protein-generated sulfates activate PI3K/Akt via their receptors.	[56, 58, 59]
Carbohydrates			
Glucose	Humans with diabetes, rat extensor digitorum longus muscle, mouse cardiac fibroblasts.	Cause insulin response; insulin resistance and stage 2 of the kinase insensitivity (Table 1); modulate PI3K/Akt/GSK3 activities; add inflammation and apoptosis.	[61–63]
Fructose	SD rats with diabetes, mouse hepatocytes.	Increase NF-κB activity which associates with GSK3 activity.	[96, 97]
D-galactose	Mice, human neuroblastoma cells.	Activate caspase-3, which associates with GSK3/p53 binding.	[15, 98]
Polysaccharides	Rats with diabetes, cancer patients, C57BL/6 mice [68, 99], human hepatocellular carcinoma, human melanoma cells, human osteosarcoma, human gastric carcinoma cells, Balb/c mice, T2D rats, human hepatocellular carcinoma cells,^a^ KKAy mice.	Modulate PI3K/Akt and/or GSK3 activities.	[68, 99–107]
Fats			
Intracellular lipid	Human embryonic kidney cells, human monocytes, mouse embryonic fibroblasts.	Modulate GSK3/PLIN2 association, GSK3 activity, expression of GSK3 substrates and cell growth/survival, and increase pYGSK3 levels (long-term).	[6]
Extracellular lipid including palmitic acid	Human hepatocellular carcinoma cells, normal men.	Generate insulin resistance and stage 3 of the kinase insensitivity (Table 1) and decrease insulin-induced PI3K activity.	[67, 68]
Sterol including androgen	Human prostate cancer epithelial cells.	Increase Akt activity.	[69]
Monoacylglycerol	Mouse neural crest cells.	Activate PI3K.	[70]
Diacylglycerol and medium-chain triacyglycerol	Human breast cancer cells, human brain glioblastoma cells, human alveolar basal epithelial cells, livers of malnourished Wistar rats.	Activate Akt.	[71, 72]
High-fat diet	C57BL16 mice, Tg2576 mice, diabetes- and obesity-prone C57BL/6 J mice, C57BL/6J mice.	Induce insulin insensitivity which can be improved by overexpression of PLIN2, increase glucose intolerance and insulin resistance, and decrease PI3K/Akt activities and raise GSK3 activity, stage 3 of the kinase sensitivity (Table 1), whereas glucose metabolism can be ameliorated if GSK3 activity is inhibited.	[33, 43, 73, 74]
High lipid levels	Mouse myoblast cells.	Overexpression of PLIN2 betters insulin sensitivity reduced by fatty acids.	[33]

^a^KKAy mice: The KK-*Ay* mouse is a T2D model that exhibits marked obesity, glucose intolerance, severe insulin resistance, dyslipidemia, and hypertension

The interplay between dietary glucose and body insulin response has been well elucidated, and the impairment of insulin-triggered PI3K/Akt signaling is the underlying mechanisms for T2D [61]. Under certain conditions, decreased activation of Akt but not PI3K can occur in skeletal muscles of diabetic rats [62], a situation designated as stage 2 of the kinases insensitivity (Table 1). Moreover, while short-term hyperglycemia activates PI3K/Akt and suppresses GSK3 [62], prolonged hyperglycemia (as in diabetics) can lead to inflammation and apoptosis associated with high GSK3 activity [14–16, 63, 64].

Hyperlipidemia (i.e. high levels of extracellular lipids) and obesity (i.e. high levels of intracellular lipids) can increase GSK3 activity via (i) suppression/overstimulation of the PI3K/Akt pathway and (ii) dissociation of GSK3/PLIN2. High extracellular lipids, such as fatty acids, can modulate GSK3 activity via the fatty acid receptor (GPCR) [65] and activation of PI3K/Akt [66]. However, extended high fatty acid concentrations induce insulin resistance by decreasing PI3K activation [67], a situation defined as stage 3 of the kinase insensitivity (Table 1). Although pSGSK3 levels do not differ in cells treated without or with oleic acid [6], cells treated with palmitic acid exhibit increased Akt/PI3K/GSK3 insensitivity [68], also showing the condition of stage 3 of the kinase insensitivity (Table 1). Sterol (including androgen) [69], monoacylglycerol [70], diacylglycerol [71] and medium-chain triacylglycerol [72] all exert an effect on PI3K/Akt.

High level of intracellular lipids, as discussed above, exerts an effect on GSK3 via PLIN2; short-term lipid accumulation increases GSK3/PLIN2 association, whereas long-term lipid accumulation decreases GSK3/PLIN2 association [6]. GSK3 in oleic acid treated cells is activated because of an increased level of pYGSK3 and dissociation of GSK3 from PLIN2 [6]. In vivo studies show that unlimited high-fat diet is associated with low PI3K/Akt and high GSK3 activities, stage 3 of the kinase insensitivity (Table 1) in mouse brains [43]. High fat diet-induced GSK3 activity is linked with development of insulin resistance and T2D in obesity-prone mice [73], so that suppression of GSK3 activity betters insulin-induced glucose metabolism in mice fed high-fat diet [74]. Overexpression of PLIN2 can raise insulin sensitivity in skeletal muscle despite high lipid levels [33], which is probably via high PLIN2 expression-gained IRS1 expression regardless of lipid levels [6]. The same event [6] can also happen in vivo where PLIN2 overexpression attenuates insulin insensitivity induced by high-fat diet [33].

**Fig. 1:**
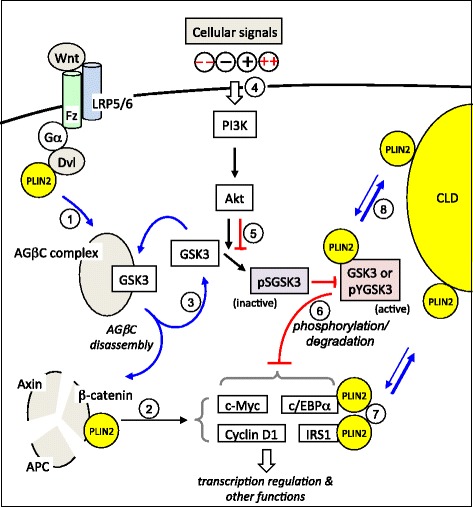
Dual regulation of GSK3 by the PI3K/Akt/GSK3 pathway and PLIN2. Upon Wnt stimulation, the axin-GSK3-β-catenin complex (AGβC) is disassembled and the process is dependent of PLIN2 (denoted by *curvy blue arrows*) [1]. The released β-catenin from the AGβC complex activates transcription of factors involved in cell growth/survival (e.g. c-Myc, c/EBPα, and cyclin D1) or in insulin signaling (e.g. IRS1) [2], whereas the released GSK3 from the AGβC complex can be present in cytosol or recycle back to the AGβC complex [3]. The GSK3 activity is regulated mainly through the PI3K/Akt pathway that relays extracellular and intracellular (not shown) signals; − and + denote normal inhibitory and stimulatory signals, respectively, whereas −− and ++ (in *red*) denote over-inhibitory (e.g. the potency of the LY compounds used in (Ref 216)) and over-stimulatory (*e.g.* the potency of VEGF used in (Ref 5)) signals, respectively [4]. Different stages of kinase insensitivity and uncontrolled GSK3 activation are summarized in Table 1. Under − and −− conditions, the respective pSGSK3 levels are reduced but GSK3/pYGSK3 levels are increased (denoted by *red blockage* between the two) [5], perhaps inducing moderate and high activities of GSK3, respectively. Under ++ conditions, all the kinases tend to become insensitive (denoted by *red blockages*), generating unbridled GSK3 activity (Table 1) that can phosphorylate its substrates and render their degradation [6]. Accumulation of cytosolic lipid droplets (*CLD*) alters the association of PLIN2 and its binding proteins such as Dishevelled (*Dvl*), β-catenin, c/EBPα, and IRS1 [7]. The *bold blue lines* denote high affinity between PLIN2 and CLD [8]. LRP5/6: lipoprotein receptor-related proteins 5/6; Fz, frizzled; Gα, guanine nucleotide binding protein α subunit

In a broader sense, nutrients also include minerals (Table 3), vitamins (Table 4), food supplements such as antioxidants (Table 5), condiments and ingredients in drinks (Table 6). All of these nutrients, taken up as foodstuff, represent the major environmental factor that influences activities of the PI3K/Akt/GSK3 axis. There are two major types of such environmental factors that can unlock the respective inhibitory effects of PLIN2 and PI3K/Akt on GSK3. One is intracellular lipid that sequesters PLIN2 [6], which may provide a mechanism for the etiology of diseases such as Alzheimer’s disease, CVD and T2D that are often associated with obesity. The other is via PI3K/Akt inhibition or overstimulation. High doses of certain vitamins can suppress PI3K/Akt activities [75–77], playing similar roles to that of LY compounds (PI3K inhibitors) [78], which can be defined as over-inhibition events (Fig. 1) since those reagents generate severe physiological effects via suppression of PI3K [75–77], or over-stimulate the pathway [79–81], leading to augmented GSK3 activity with increased risks of mortality [82]. Dampening a food ingredient-induced PI3K/Akt activation [63] or maintaining a hormone-caused unregulated GSK3 activity in regulated ranges [83], antioxidants can buffer the effects (overstimulation) generated by the abnormal factors on the pathway.

**Table 3:** Minerals alter PI3K/Akt and/or GSK3 activities

Minerals	Model system	Observed effects	Ref.
High levels in the body			
Sodium, chloride, potassium	Monkey kidney cells, HeLa cells, human or mouse melanoma, mouse renal distal convoluted tubule cells, ^a^Wnk4^+/+^ and Wnk4^D561A/+^ mice, male SD rats.	High salt foods (mainly NaCl) cause potential hyperosmotic stress, which modulates PI3K/Akt/GSK3 activities; increase or decrease phosphorylation of NaCl transporter, regulated via insulin/PI3K pathway by low salt diet or high salt diet; high salt food causes early insulin resistance, stage 0 of the kinase insensitivity (Table 1).	[24, 49–52, 108, 109]
Calcium	Mouse osteoblast, human thyroid cancer cells, mouse neural crest cells.	Exert effects on PI3K/Akt and/or GSK3 pathway.	[47, 48, 110]
Manganese sulfate	Mouse macrophages.	Anti-inflammation via PI3K/Akt.	[111]
Magnesium sulfate	Rats with intestinal ischemia-reperfusion injury.	Protect injury via PI3K/Akt.	[58]
Fucosylated chondroitin sulfate	T2D mice.	Improve insulin sensitivity via activation of PI3K/Akt.	[59]
Heparan sulfate	Human normal astrocytes, and malignant gliomas, human breast cancer cells, human umbilical vein endothelial cells, wild type and Syndecan-1−/− mice infected by influenza.	Increase/Reduce PI3K/Akt/ERK signaling, carcinogenesis/anti-cancer and anti-inflammation.	[112–115]
Magnesium	Brains of Wistar rats, patients with diabetes.	Required for GSK3 activation; EDTA Chelation Therapy decreases CVD events in patients with diabetes.	[116, 117]
Trace levels in the body	Wistar rats, mouse hepatocytes.	Induce injury regulates PI3K/Akt/GSK3β pathway, whereas aged rats have less sensitivity of the regulation; iron oxide nanoparticles-mediated cytotoxicity related to PI3K/Akt pathway.	[118, 119]
Iron			
Zinc or copper	Mouse myogenic cells, monkey kidney cells, mouse embryonic fibroblast, human hepatoma cells, human neuroblastoma cells, human prostate epithelial cells.	Stimulates PI3K/Akt signaling, leading to inhibition of GSK3β; zinc deficiency adds Akt signaling.	[120–124]
Iodine	SD rats.	Required for synthesis of thyroid hormones that activates Akt.	[22]
Manganese	Mouse microglial cells, human lung epithelial cells.	Induce inducible nitric oxide synthase expression via activation of both MAP kinase and PI3K/Akt pathways; increase the expression of prostaglandin-endoperoxide synthase 2 (COX-2) via p38 and PI3K/Akt.	[125, 126]
Zinc and manganese	South Hampshire and Merino^b^ CLN6 sheep.	Increased in the model with reduced expression of ceroid-lipofuscinosis neuronal protein 6, accompanying with activation of Akt/GSK3 signaling (stage 1 of the kinase insensitivity (Table I)), and neurodegeneration.	[127]
Selenium	Human prostate cancer cells.	Reduce the activities of PI3K/Akt.	[128]
Aluminum fluoride	Mouse adipose cells.	Induce G-protein-linked PI3K signaling.	[129]
Fluorine	SD rats.	Accumulation of it relates to increase of PI3K/Akt and p38 and tissue in bone tissue of fluorosis rats.	[130]
Chromium	Mouse myoblast cells.	Increase expression glucose transporter and insulin receptor, resulting in enhanced glucose uptake.	[131]

^a^ *WNK* with-no-lysine kinase,^b^ *CLN* ceroid-lipofuscinosis neuronal protein

**Table 4:** ^a^Vitamins change PI3K/Akt and/or GSK3 activities

Vitamins	Model system	Observed effects	Ref.
Vitamin A	Mouse embryonic stem cells, human neuroblastoma cells, human bronchial epithelium.	PI3K via IGF-1 receptor/IRS1; suppress cyclin D1 protein expression via GSK3, stage ≥ 1 of the kinase insensitivity (Table 1).	[79–81]
Vitamin B1 analog	Mice with diabetes, mouse cadiomyocytes, human embryonic kidney cells, ^b^APP/PS1 mice.	Activate Akt, preventing diabetes-induced diastolic dysfunction and heart failure; avert high glucose-induced β-amyloid related to GSK3 activity; inhibit GSK3 activity to subdue cognitive damages and beta-amyloid accumulation.	[132–134]
Vitamin B3 (Niacin) and vitamin B6 (pyridoxine)	Human epidermoid carcinoma cells, Chinese hamster ovary cells, ^c^Hca_2_^+/−^ mice, human platelets.	Augment PI3K/Akt activities.	[135, 136]
Vitamin B8 (inositol)	Smokers.	Suppress Akt and ERK.	[137]
Vitamin B9 (folic acid)	Mouse neural stem cells.	Stimulate cell growth by modification of epigenetics of PI3K/Akt/cAMP response element-binding protein pathway.	[138]
Vitamin B10 (para- aminobenzoic acid)	Zebrafish embryos.	Raise pSGSK3β reduced by valproic acid, an anti-epilepic drug).	[139]
Vitamin B11 (salicylic acid)	Human umbilical vein endothelial cells and human foreskin fibroblasts, murine myoblasts, Humans with inflammation.	Inhibit COX-2 gene transcription, resulting in anti-inflammatory effects.	[140–142]
Vitamin B13 (orotic acid)	Human umbilical vein endothelial cells, SD rats.	Patients with orotic acid metabolic disorders may reduce insulin response and PI3K/Akt signaling, generating insulin resistance.	[143]
Vitamin B14	Human bone marrows.	Increase cell growth and haemopoiesis.	[144]
Vitamin B17 (amygdalin)	Human bladder cancer cells.	Inhibit cell growth via activated Akt-related pathways.	[145]
Pyrroloquinoline quinine	Rat cardiomyocytes, hippocampal neurons and brain cortex from SD rats.	Possibly naturally existing in vitamin B complexes can activate PI3K/Akt and reduce cell apoptosis or inhibits GSK3β activity in nervous tissues of glutamate-injected animals.	[146–148]
Vitamin C or vitamin E	Human colon cancer cells.	Inhibit casein kinase 2 (CKII) downregulation-mediated aging in cells, whereas suppression of CKII raises PI3K/Akt activities.	[149]
Vitamin C	Human breast cancer cells.	Enhance a synthetic anti-cancer drug, mitoxantrone-induced cytotoxicity.	[150]
Vitamin D		Vitamin D receptor mediates PI3K/Akt activation; vitamin D reduces caspase activities for cell apoptosis via vitamin D receptor/PI3K/Akt pathway.	[151, 152]
	Human myeloid leukaemic cells, rat osteoblasts.	Vitamin D deficiency induces hyperinsulinemia and insulin resistance in obese mice.	[153]
	C57BL/6J mice.	Enhance effects of PI3K inhibitors on cell growth.	[154]
	Human prostate cancer cells.	Induce the tolerance or immunosuppression through the PI3K/Akt pathway.	[155, 156]
	Human monocyte-derived tolerogenic dendritic cells, human CD3+ T cells.	Activate MAP kinase and/or PI3K/Akt for protecting cell death.	
Vitamin E	Cultured mouse cortical neurons, human neuroblastoma.		[157, 158]
	Human breast cancer cells, human prostate cancer cells.	Tocotrienols (natural forms of vitamin E) or tocopherol (the saturated form of vitamin E)-associated protein can suppress cancer growth via inhibition of PI3K.	[75, 76]
	Mouse neoplastic mammary epithelial cells.	Gamma-tocotrienol can block human epidermal growth factor receptor 3-dependent PI3K/Akt mitogenic signaling.	[77]
Vitamin J (catechol)	Mouse microglial cells.	Iridoid and catechol (vitamin J) derivatives of natural products, have anti-inflammatory activities via inhibition of the PI3K/Akt and p38 pathways.	[159]
Vitamin K	Apoptotic cells.	Protein Gas6 and S are vitamin K dependent proteins and ligands of RTK that can regulate PI3K/Akt pathway.	[160]
Vitamin P	Mouse primary neurons.	Increase PI3K/Akt activities and the survival of motoneurons via tropomyosin-receptor kinase B.	[161]
Vitamin U	Mice, rats.	Vitamin U (methylmethioninesulfonium chloride) reduces capillaries’ permeability of animal skin; protecting gastric mucosa from lesion caused by aspirin, an acetylated form of salicylic acid (vitamin B11) with anti-inflammatory effects.	[162]

^a^Vitamins: not all the vitamins are widely accepted as vitamins, ^b^ *APP/PS1* amyloid precursor protein/presenilin-1, ^c^ *Hca2* niacin receptor 1

**Table 5:** Antioxidants influence PI3K/Akt and/or activities

Antioxidants	Model system	Observed effects	Ref.
Anthocyanidins	Hypercholesterolaemic patients, human stomach cancer cells, human breast cancer cells, human hepato-carcinoma cells.	Suppress PI3K/Akt signaling pathway via epidermal growth factor receptor pathway, or levels of pSGSK3β and β-catenin in a tumor xenograft model.	[163–166]
Mulberry anthocyanidin	Human liver cancer cells.	Activate PI3K/Akt.	[167]
Berberine	Human melanoma cells, SD rats.	Inhibit PI3K/Akt and/or GSK3β activities.	[52, 168]
	Murine neural crest cells, murine primary neurons, mice with cerebral and reperfusion, human chondrosarcoma cells.	Increase PI3K/Akt activities and cell growth/survival in other studies.	[169, 170]
Curcumin	Human Burkitts’ lymphoma, human esophageal cancer cells, human renal cancer cells.	Enhance radiation- or PI3K/Akt inhibitors-induced or directly induce apoptosis by suppression of PI3K/Akt signaling pathway.	[171–173]
	Rat cardiomyocytes, human prostate cancer cells, Balb/c mice.	Protect cells from apoptosis induced by a high glucose level via upregulation of Akt/GSK3β serine/threonine phosphorylation levels via protein phosphatase-dependent mechanism or inhibits GSK3β activity in vitro or in vivo.	[174–176]
Ergosterol	Streptozotocin-induced diabetes in mice, human cancer cells.	Restore PI3K/Akt signaling damaged in diabetic mice; ergosterol-related compounds induce cell apoptosis depending on a protein-promoted Akt activation.	[177, 178]
Garlicin	Human cellosaurus cells	Suppress PI3K/Akt pathway.	[179]
Garlic	Fructose-fed diabetic SD rats	Activate PI3K/Akt in Diabetes rats.	[96]
Luteolin	Human epidermoid carcinoma cells and their murine cells xenograft model, human umbilical vein endothelial cells, human prostate cancer cells, human colon cancer cells, human glioblastoma cells.	Inhibit VEGF-increased PI3K/Akt activities or IGF-1-increased the phosphorylation levels of PI3K/Akt/GSK3 or down-regulate PI3K/Akt pathway.	[180–183]
	Cardiomyocyte in rats with ischemia/reperfusion, murine neural crest cells.	Decrease apoptosis via PI3K/Akt pathway in a rat model or persistently activate Akt in cells.	[184, 185]
Lycopene	Prostate epithelial cells.	Inhibit IGF-1-induced Akt/GSK3 serine/threonine phosphorylation levels.	[83]
	Patients, human prostate cells.	Its effects on PI3K/Akt pathway are inhibitory in prostate cancer.	[186]
Phytoestrogens	Human embryonic kidney cells, mouse preosteoblastic cells.	Increase phosphorylation levels of Akt and GSK3β as well as the Wnt/β-catenin signaling.	[187]
Isoflavones	Human cancer cells	Inhibit PI3K/Akt signaling in cancer cells.	[188]
Soy isoflavone	SD rats with myocardial ischemia/reperfusion.	Gain PI3K/Akt pathway activities in ovariectomized rats.	[189]
Daidzein or genistein	Nude mice with various tumors	Up-regulate or down-regulate GSK3 gene/protein expression, and both belong to isoflavones.	[190]
Psoralidin	Human lung fibroblasts, mice.	A coumestan derivative suppresses pro-inflammatory cytokines and regulates PI3K/Akt pathway.	[191]
Resveratrol	Mouse cardiac fibroblasts, human glioma cells.	Inhibit high glucose-induced PI3K/Akt pathway and inflammation or reduces PI3K/Akt activities.	[63, 192]
	Neural crest cells, APP/PS1 mice.	Protect cells from apoptosis induced by high glucose via activation of PI3K/Akt pathways and increase in vivo pSGSK3β levels.	[193, 194]
Lignan including honokiol and sauchinone	Human prostate cancer cells, human myeloid leukaemic cells, mouse microphage, mouse lymphoblast, splenic lymphocytes, human glioma, breast and prostate cancer cells, human hepatocytes, WT and ^a^ *Nrf2* KO C57/BL6 mice.	Inhibits Akt signaling and generate anti-inflammatory effect via inhibition of PI3K/Akt pathway or mediate suppression of PI3K; however sauchinone, augments in vivo pSGSK3β levels.	[195–198]

^a^ *Nrf2* nuclear factor (erythroid derived 2)-like 2

**Table 6:** Condiments or ingredients in drinks modulate PI3K/Akt and/or GSK3 activities

Nutrient	Model system	Observed effects	Ref.
Condiments			
Capsaicin	^a^TRPV1-KO and wild-type C57BL/6 mice.	Exert its effect through the capsaicin receptor that is the transient receptor potential cation channel subfamily V member 1 (TRPV1).	[199]
	Human prostate cancer cells.	Regulate PI3K/Akt pathway in cultured cells and can activate microglia in mouse spine cord at a very low concentration.	[200]
	Rat spinal cord	ERK activation is detected in microglia of animal spine cord by capsaicin stimulation.	[201]
	SD rats, astrocytes and microglia from the rats, human microglia cells.	Capsaicin-activated TRPV1 mediates microglia death via calcium signaling.	[202]
	Human colorectal cancer cells.	Increase association of c/EBPβ and GSK3β, which is suggested to mediate capsaicin-induced apoptosis.	[203]
Monosodium glutamate (MSG)	Rodent striatal cholinergic interneurons.	Contain glutamate which is a non-essential amino acid and its receptor is glutamate receptor, belonging to GPCR.	[204]
	Animal nervous systems	Neurotransmitters in the brain; whether MSG clinically associates with neurologic diseases remains to be studied.	[205]
	SD rats, mouse hippocampal neuronal cells, hippocampal neurons and brain cortex from SD rats.	Induce neurodegeneration is suggested via PI3K/Akt pathway regulation and injection of glutamate into animals generates neurotoxicity via GSK3β.	[147, 148, 206]
Ingredients in drinks	Animal nervous systems.	A central nervous system (CNS) stimulant and cause biological effects via adenosine receptors that belong to GPCR.	[207–209]
Caffeine	Human neuroblastoma cells, HeLa cells, mouse neural crest cells, mouse adipocytes.	Activate PI3K/Akt pathway and prevent cell death; or induce cell apoptosis by suppressing PI3K/Akt signaling and decrease phosphorylation levels of Akt/GSK3β.	[210–212]
	Patients.	Excess caffeine can lead to caffeine intoxication (i.e. overstimulation of CNS).	[213]
Ethanol	Ethanol-induced fatty liver in mice, ^a^AA and ANA rats.	Presented in liquor can acutely induce hepatosteatosis, a process associated with PI3K/Akt activation and phosphorylation levels of Akt and GSK3β in the rat cortex.	[214, 215]
	Human vascular endothelial cells.	Low concentrations of ethanol activate PI3K/Akt signaling, inhibiting GSK3 activity, whereas high concentrations of ethanol induce caspase-3 activation and increases apoptosis	[216]
	Human cells, C57BL/6 mice.	Ethanol is metabolized to acetaldehyde by alcohol dehydrogenase in the body, and acetaldehyde is further metabolized by aldehyde dehydrogenases (ALDH).	[217, 218]
	Human hepatic stellate cells.	The acetaldehyde-enhanced gene expression requires PI3K activation.	[219–221]
	C57BL/6 mice	Ethanol administration reduces phosphorylation levels of Akt and GSK3β, which is aggravated in cardiomyocyte without ALDH-2.	[218]
Tea	Components analyzed.	Have ingredients including caffein, polyphenols and catechin containing abundant epigallocatechin gallate (EGCG).	[222]
Tea polyphenols	Mouse skin epithelial cells, human normal and keloid fibroblasts, the cultured human keloid model. Humans.	Have inhibitory effects on PI3K pathway and suppress PI3K/Akt proteins expression and/or Akt activity in vitro and in vivo in prostate cancer models, may play roles in prevention of prostate cancer.	[223–225]
EGCG	Human hepatocyte derived cellular carcinoma cells, human pancreatic carcinoma cells.	Block cell growth and induces cell apoptosis via inhibition of VEGF signaling pathway including Akt or downregulation of Akt activity.	[226, 227]
	Human alveolar basal epithelial cells, human neuroblastoma cells expressing ^b^APP-C99.	Raise cell viability by its induction of Akt activity and suppression of GSK3β activity and inhibit β-amyloid-induced neurotoxicity by suppression of GSK3β activation.	[228, 229]

^a^AA and ANA: AA (Alko, Alcohol) line of rats which prefer 10 % alcohol to water, and the ANA (Alko, Non-Alcohol) line of rats which are given only water

^b^APP-C99: an amyloid precursor protein fragment

Although GSK3 activity has not been uniformly measured in the above cited literatures, given that PI3K/Akt are the major regulators of GSK3 and that those studies present GSK3-associated phenotypes such as inflammation and apoptosis, it is not out of realm to speculate that the diet ingredients that modulate PI3K/Akt would have an impact on GSK3 activity as well. It is noteworthy that studies using the same ingredients sometime yield inconsistent or contradictory results, which could be due to differences in experimental conditions (e.g. dose or duration of the treatment and model systems chosen) that could impact the activation/inactivation of GSK3. Because unconstrained GSK3 activity can be associated with different stages of the kinases’ insensitivity (Table 1) or activated GSK3 can be caused by suppression of PI3K/Akt, it is therefore recommended all three kinases’ activities in the PI3K/Akt/GSK3 axis be determined in future studies of disease development. Measurements of mass and phosphorylation status of GSK3, GSK3-related kinases, and GSK3 substrates [4] in peripheral blood mononuclear cells (PBMC) may help diagnosis of sub-health patients [20, 84]. These measurements may also help selection of control groups in clinical studies and assessment of efficacy of treatment of diseases (e.g. HIV), because activities of GSK3-related kinases are altered by HIV infection [85–88]. Moreover, these measurements may help establishment of a relationship between GSK3 activity and various chronic and age-related disorders, including insomnia, chronic inflammation (e.g. that causes neuropathy damage) [89, 90], as well as pre-diseases/diseases signals (e.g. liver palms, obesity, abnormal face wrinkles, optic redness), among others. Thus, these measurements may supplement blood and urine tests in the surveillance of health conditions among peoples who carry genetic risks of diseases.

## Conclusions

Nature has seemingly selected GSK3 as a gatekeeper of life span. GSK3β knockout mice are systemic in apoptosis and die *in utero*, whereas GSK3 overexpression in mice also results apoptosis [91]. The question concerns whether abnormal GSK3 activity *per se* limits life span or GSK3 activity represents “the shortest side slab” in the “life span bucket” remains to be addressed experimentally, if possible. Regardless, GSK3 perhaps plays a major role in longevity [92] and in mortality incurred by infections or injury-caused inflammation. There appears to be an “uncertainty principle” governing the relationship between environmental factors and chronic diseases. Acute adverse environmental factors may not necessarily lead to diseases stages and rather create a sub-health condition. Thus, the relationship between cause and effect is not always clear. This uncertainty is also influenced by individual variations. Despite the complexity of cellular signaling pathways and their intrinsic cross talks, the one thing that seems certain is the convergence of high GSK3 activity under all adverse conditions. In this regard, GSK3 activity is inhibited by a short-term stimulus but released by a long-term one (e.g. under oleic acid treatment conditions), and only the increased GSK3 activity is consistent with the observation of metabolic disorders.

The presence of the dual mechanism (i.e. PI3K/Akt-mediated phosphorylation and lipid-mediated GSK3/PLIN2 interaction) for GSK3 regulation provides a link between energy homeostasis and cellular functionality. Whether or not there is a cross talk between the two regulatory pathways remains to be determined. Palmitic acid-treated cells show decrease in Akt/PI3K/GSK3 sensitivity [68], which is a good lipid model to study the crosstalk between the two pathways.

Development of drugs targeting GSK3 inhibition is recently making progress [9, 93], including synthetic inhibitors that delay progress of diseases [10, 94, 95] and natural inhibitor such as lithium that can extend Drosophila lifespan by 16 % [92]. However, high concentration of lithium appears to be toxic [92] and over-suppression of GSK3 activity increases the risk of developing cancer [6, 10]. It merits further investigation to ascertain whether or not GSK3 inhibition could be a druggable target for clinical treatments.

To conclude, the overstimulation-induced kinase insensitivity that leads to uncontrolled GSK3 over-activation represents a key cellular/molecular mechanism that is intimately associated with aging and many age-related chronic diseases. As an ancient Taoism saying goes, *the extremity reached, the course reversed* (物极必反). Factors contributing to such an overstimulation range from life style (unbalanced diets), habit and behavior (including medication) to psychological conditions (including negative emotions or ecstasy, an extremely positive emotion). Therefore, preventing from over-activation of GSK3 in the body, through adoption of healthy habits/lifestyles including balanced diets and a positive attitude, does a great favor to our health.

## Abbreviations

AA and ANA, AA (Alko, Alcohol) line of rats which prefer 10 % alcohol to water, and the ANA (Alko, Non-Alcohol) line of rats which are given only water; ADRP, adipose-differentiation related protein; AGβC, axin/GSK3β/β-catenin complex; AIDS, acquired immune deficiency syndrome; ALDH, aldehyde dehydrogenases; APP/PS1, amyloid precursor protein/presenilin-1; APP-C99, an amyloid precursor protein fragment; c/EBP, CCAAT enhancing binding protein; CK II, casein kinase 2; CLN, ceroid-lipofuscinosis neuronal protein; CNS, central nervous system; COX-2, cyclooxygenase-2, prostaglandin-endoperoxide synthase 2; CVD, cardiovascular diseases; Dvl2, dishevelled 2; EDTA, ethylenediaminetetraacetic acid; EGCG, epigallocatechin gallate; ERK, extracellular signal-regulated kinases; Fz, Frizzled; Gas6, growth arrest-specific 6; GPCR, G-protein coupled receptors; GSK3, glycogen synthase kinase 3; Gα, guanine nucleotide binding protein α subunit; Hca2, Niacin receptor 1; HIV, human immunodeficiency virus; HRPE, human retinal pigment epithelial cells; IGF-1, insulin-like growth factor 1; IL17RC, interleukin 17 receptor C; IRS1, insulin receptor substrate 1; KKAy mice, The KK-*Ay* mouse is a type 2 diabetic model that exhibits marked obesity, glucose intolerance, severe insulin resistance, dyslipidemia, and hypertension; LRP, lipoprotein receptor-related proteins; MSG, monosodium glutamate; NaCl, sodium chloride; NF-κB, nuclear factor kappa-light-chain-enhancer of activated B cells; *Nrf2*, nuclear factor (erythroid derived 2)-like 2; NSF, *N*-ethylmaleimide-sensitive factor; PBMC, peripheral blood mononuclear cells; PDK1, phosphoinositide-dependent kinase-1; PI3K, phosphoinositide 3-kinase; PK, protein kinase; PLIN, perilipin; Protein S, S-protein; pSGSK3, serine- phosphorylated form of GSK3; pYGSK3, tyrosine-phosphorylated form of GSK3; RTK, receptors of tyrosine kinase; SD, Sprague Dawley; siRNA, short interfering RNA; SNAP, soluble NSF attachment protein; SNARE, SNAP (soluble NSF attachment protein) receptor; T2D, type 2 diabetes; TCF, T-cell factor; TRPV1, transient receptor potential cation channel subfamily V member 1; VEGF, vascular endothelial growth factor; WNK, with-no-lysine kinase
